# Per-Arnt-Sim Kinase (PASK) Deficiency Increases Cellular Respiration on a Standard Diet and Decreases Liver Triglyceride Accumulation on a Western High-Fat High-Sugar Diet

**DOI:** 10.3390/nu10121990

**Published:** 2018-12-15

**Authors:** Jenny A. Pape, Colleen R. Newey, Haley R. Burrell, Audrey Workman, Katelyn Perry, Benjamin T. Bikman, Laura C. Bridgewater, Julianne H. Grose

**Affiliations:** 1 Department of Microbiology and Molecular Biology, Brigham Young University, Provo, UT 84602, USA; jennyapattison@gmail.com (J.A.P.); colleennewey@byu.edu (C.R.N.); haley.burrell@gmail.com (H.R.B.); workm050@umn.edu (A.W.); laura_bridgewater@byu.edu (L.C.B.); 2 Department of Physiology and Developmental Biology, Brigham Young University, Provo, UT 84602, USA; kate.perry22@gmail.com (K.P.); benjamin_bikman@byu.edu (B.T.B.)

**Keywords:** PAS kinase, PASK, high-fat high-sugar diet, western diet, respiration, triglycerides, lipids, liver, hepatic, lipidomics, electron transport chain, female, mice, sexual dimorphism

## Abstract

Diabetes and the related disease metabolic syndrome are epidemic in the United States, in part due to a shift in diet and decrease in physical exercise. PAS kinase is a sensory protein kinase associated with many of the phenotypes of these diseases, including hepatic triglyceride accumulation and metabolic dysregulation in male mice placed on a high-fat diet. Herein we provide the first characterization of the effects of western diet (high-fat high-sugar, HFHS) on Per-Arnt-Sim kinase mice (PASK^−/−^) and the first characterization of both male and female PASK^−/−^ mice. Soleus muscle from the PASK^−/−^ male mice displayed a 2-fold higher oxidative phosphorylation capacity than wild type (WT) on the normal chow diet. PASK^−/−^ male mice were also resistant to hepatic triglyceride accumulation on the HFHS diet, displaying a 2.7-fold reduction in hepatic triglycerides compared to WT mice on the HFHS diet. These effects on male hepatic triglyceride were further explored through mass spectrometry-based lipidomics. The absence of PAS kinase was found to affect many of the 44 triglycerides analyzed, preventing hepatic triglyceride accumulation in response to the HFHS diet. In contrast, the female mice showed resistance to hepatic triglyceride accumulation on the HFHS diet regardless of genotype, suggesting the effects of PAS kinase may be masked.

## 1. Introduction

Diabetes and the related disease metabolic syndrome are an ever-increasing epidemic in today’s society. In 2015, 9.4% of the United States population had diabetes and the estimate for metabolic syndrome was much higher at 30% [1,2,3]. Characterized by having a combination of increased blood pressure, high blood sugar, excess body fat around the waist, and abnormal triglyceride levels, metabolic syndrome increases one’s risk for heart disease, stroke, and diabetes [1]. The increasing rates of these diseases are in part due to a global shift toward energy-dense, high-fat, low nutrient foods, combined with a decrease in physical activity [4]. As these changes affect the body as a whole, they also challenge the cellular processes in the body as it attempts to adapt to the new nutrient and activity levels. Nutrient sensors play a critical role in adapting to these new levels by constantly monitoring cellular nutrients and regulating cellular pathways to maintain homeostasis [5,6]. When dysregulation in nutrient-sensing pathways occurs, many human diseases such as diabetes and metabolic syndrome develop.

PAS kinase is a nutrient-sensing protein kinase that is conserved from yeast to man [7]. It has been reported to regulate many of the phenotypes associated with metabolic syndrome and/or diabetes. PAS kinase deficiency decreases insulin production, insulin resistance, body weight, and hepatic triglyceride accumulation, while leading to increased glycogen storage as well as metabolic rate (for research articles and associated recent reviews see [8,9,10,11,12,13,14,15,16,17,18,19,20,21]). For example, PAS kinase-deficient (PASK^−/−^) male mice are resistant to weight gain, hepatic triglyceride accumulation and insulin resistance when placed on a high-fat (HF) diet [18]. Without a change in food-intake or exercise, these PASK^−/−^ male mice also exhibit a hypermetabolic phenotype, giving off more CO_2_ and taking in more O_2_. In addition, several mRNA’s involved in lipid biosynthesis are downregulated in PASK^−/−^ male mice such as stearoyl-CoA desaturase 1 (SCD1), long-chain fatty-acid elongase, fatty-acid transporter (CD36) and the lipid-responsive nuclear hormone receptor peroxisome proliferator-activated receptor γ. This PAS kinase-dependent decrease in hepatic triglycerides on a HF diet has been confirmed in Sprague-Dawley rats treated with pharmacological inhibitors [13]. Furthermore, the genetic and pharmacological inhibition of PAS kinase in cultured cells suggests that these effects are in part due to the inhibition of SREBP-1c proteolytic maturation [13]. PAS kinase itself is also regulated by cellular nutrient status. PAS kinase activity and/or mRNA expression increases under conditions of increasing nutrients, specifically upon feeding in mice [13] or at high glucose concentrations in mammalian cells [14].

Herein we characterize the molecular effects of PAS kinase on respiration and triglyceride metabolism, as well as how PAS kinase alters these pathways in response to diet and sex. This study utilized cellular respiration assays, western blots for electron transport chain protein abundance, and triglyceride metabolomics approaches. To determine the effect of diet in PASK^−/−^ mice, we investigated the addition of sugar to the previously reported HF diet (a high-fat high-sugar diet, HFHS diet). This diet provides conditions where PAS kinase may be more active (high-sugar) while also more accurately reflecting the Western Diet of today’s society (high-sugar and high-fat [22]). In addition, we consider sex (female versus male mice) when characterizing the molecular effects of PAS kinase, which has been not been previously reported. These findings will aid in understanding the effects of PAS kinase as well as a HFHS diet on metabolism, shedding light on the pathways that contribute to diseases such as metabolic syndrome and diabetes.

## 2. Materials and Methods

Animals: C57BL/6 (Charles River Laboratories Wilmington, MA, USA) PASK^−/−^ mice were generously donated by Jared Rutter (University of Utah) and were described previously [18]. Wild type C57BL/6 were obtained from Charles River Laboratories Wilmington, MA. Age-matched male and female wild type and PASK^−/−^ mice, generated by breeding PASK^+/−^ mice, were placed on a HFHS diet (D12266Bi Condensed Milk Diet from Research Diets—16.8% kcal protein, 31.8% kcal fat, 51.4% kcal carbs—primarily sucrose, lactic casein and corn starch) or normal chow (NC) diet (Teklad Rodent Diet 8604 from Envigo—32% protein, 14% fat, 54% carbs—primarily dehulled soybean meal) at 12 weeks old and maintained on the diet for a total of 25 weeks. Mice were co-housed at no more than 5 mice/cage according to sex, genotype and assigned diet in a conventional animal house. Food and water were freely available, and mice were on a 12-h light/dark cycle. All procedures were approved by the Brigham Young University Institutional Animal Care and Use Committee (protocol numbers 13-1003 submitted by L.C.B.).

Study design: The design was based on the characterization of PASK^−/−^ male mice on the HF diet wherein mice were placed on the HF diet at 12 weeks, and 12–24 mice of each group were used to obtain statistical significance of several phenotypes including body weight [18]. The study herein contains 8 experimental groups including male and female WT NC diet, WT HFHS diet, PASK^+/−^ NC diet, PASK^+/−^ HFHS diet that were kept on the HFHS diet for 25 weeks prior to tissue harvest. More male then female mice were required due to limited tissues (such as liver tissue for triglyceride assay versus oxygen consumption assay). An account of all animals used in this study is provided in Figure S1.

Respiration assays: Liver tissue and soleus muscle was harvested and immediately used for respiration assays. O_2_ consumption was determined using an O2K oxygraph (Oroboros Instruments Corp, Innsbruck, Austria) as previously described [23]. Tissues were minced with a scalpel and permeabilized with saponin (50 ug/ml). A baseline respiration rate was determined in each respiration chamber and then the samples were added. Respiration was measured by following the substrate-uncoupler-inhibitor-titration (SUIT) protocol: glutamate, malate, and succinate (GMS) were added to assess complex I and II electron flow. ADP (2.5 mM) was then added to determine oxidative phosphorylation capacity (GMSD). Following data collection, three-factor Analysis of Variance (ANOVA) was performed using JMP Pro14 software with Tukey post-hoc test for three-factor and two-factor comparisons and students t-test for one-factor comparisons.

Western blot analysis: Muscle tissue was homogenized using the Bullet Blender Storm 24 (Next Advance) in RIPA Lysis and Extraction buffer (ThermoFisher Scientific, Waltham, MA, USA catalog number 89900) with Halt Protease Inhibitor Cocktail (ThermoFisher Scientific, Waltham, MA, USA, catalog number 78438) using 2 mm Zirconium oxide beads. Protein concentration was determined using the Pierce Coomassie Plus (Bradford) Assay Reagent (ThermoFisher Scientific, Waltham, MA, USA). An equal amount of protein (2 ug) was loaded on a 12% SDS-PAGE gel, separated, then transferred onto a nitrocellulose membrane. After incubation with 5% nonfat milk in tris-buffered saline with Tween 20 (TBST), the membrane was rinsed 2 times with tris-buffered saline (TBS) and then probed with the OxPhosBlue Native WB Antibody Cocktail (ThermoFisher Scientific, Waltham, MA, USA) containing mouse monoclonal NDUFA9, SDHA, UQCRC2, COX IV, and ATP5A antibodies for 2 days (these correspond to subunits of complex I, II, III, IV and V). Membranes were rinsed twice with TBST, once with TBS then incubated with a 1:1000 dilution of horseradish peroxidase-conjugated anti-mouse antibodies for 2 h. Blots were rinsed and then developed using the WesternBright ECL HRP substrate (Advansta Inc., San Jose, CA, USA, catalog number K-12045-D50) according to the manufacturer’s protocol. Bands were quantified using the ImageJ software version 1.50i (National Institute of Health, Bethesda, MD, USA) [24]. Two-factor ANOVA was performed using JMP Pro14 (version 14.0) software (SAS Institute, Cary, NC, USA) with student’s t-test for one-factor analysis.

Triglyceride assays: Mouse liver samples were homogenized in 110 µL of PBS—Triton. Hepatic triglyceride levels were measured using the BioVision (Milpitas, CA, USA) Triglyceride Quantification Colorimetric/Fluorometric Kit (K622) according to manufacturer’s protocol, and absorbance was measured at 530–590 nm. Protein concentration was determined using the Pierce Coomassie Plus (Bradford) Assay Reagent (ThermoFisher Scientific, Waltham, MA, USA). Three-factor ANOVA was performed using JMP Pro14 software with Tukey post-hoc test for three-factor and two-factor interaction analysis and students t-test for one-factor analysis.

Liquid chromatography-mass spectrometry (LC/MS) lipidomics: Forty-four triglycerides were analyzed by LC/MS at the University of Utah Metabolomics Core Facility. Triglycerides were extracted from frozen tissue in 225 µL ice-cold MeOH containing internal standards (Avanti 860902 TG (16:0/18:1/16:0-d5) 100 µg/mL, 10 µL each/sample; cholesterol-d7, 100 µg/mL, 20 µL each/sample) 750 µL of ice-cold MTBE (methyl tert-butyl ether) in bead mill tubes (1.4 mm ceramic, QIAGEN, Venlo, Netherlands, catalog number 13113-50). The sample was homogenized in one 30 s cycle, rested on ice for 15 min, then 300 µL of water was added to induce phase separation. Samples were then centrifuged at 20,000 g for 5 min at 4 °C, the upper phases are collected separately and evaporated to dryness under vacuum. Triglyceride samples are reconstituted in 200 µL ACN:H2O:IPA (1:1:2) + 0.1% formic acid and transferred to an LC/MS vial with insert for analysis. A pooled QC sample were prepared by taking 40 µL aliquots from each sample. Concurrently a process blank sample was brought forward. Triglyceride extracts were separated on a Waters (Milford, MA, USA) Acquity UPLC CSH C18 1.7 µm 2.1 × 100 mm column maintained at 60 °C connected to an Agilent (Santa Clara, CA, USA) HiP 1290 Sampler, Agilent 1290 Infinity pump, equipped with an Agilent 1290 Flex Cube and Agilent 6530 Accurate Mass Q-TOF dual ESI mass spectrometer. For positive mode, the source gas temperature was set to 200 °C, with a gas flow of 11 L/min and a nebulizer pressure of 50 psig. VCap voltage was set at 5000 V, fragmentor at 100 V, skimmer at 85 V and Octopole RF peak at 750 V. For negative mode, the source gas temperature was set to 270 °C, with a drying gas flow of 8.5 L/min and a nebulizer pressure of 40 psig. VCap voltage is set at 3000 V, fragmentor at 247.5 V, skimmer at 57.5 V and Octopole RF peak at 750 V. Reference masses in positive mode (m/z 121.0509 and 922.0098) were infused with nebulizer pressure at 2 psig, and in negative mode (1033.988, 966.0007, 112.9856 and 68.9958) were infused with a nebulizer pressure at 5 psig. Samples were analyzed in a randomized order in both positive and negative ionization mode in separate experiments acquiring with the scan range between m/z 100 and 1700. Mobile phase A consisted of ACN:H2O (60:40 v/v) in 10 mM ammonium formate and 0.1% formic acid, and mobile phase B consisted of IPA:ACN:H2O (90:9:1 v/v) in 10 mM ammonium formate and 0.1% formic acid. The chromatography gradient for both positive and negative modes started at 15% mobile phase B then increased to 30% B over 2 min, it then increased to 52% B from 2 to 2.5 min, then increased to 82% B from 2.5 to 11 min, then increased to 95% B from 11 to 11.5 min, then increased to 99% B from 11.5 to 13.5 min. From 13.5 to 20 min it was held at 99% B, then decreased to 15% B from 20 to 20.2 min and held there from 20.2 to 25 min. Flow was 0.35 mL/min throughout, injection volume was 1 µL for positive mode and 5 µL for negative mode. Tandem mass spectrometry was conducted using the same LC gradient at a collision energy of 40 V. Results from LC/MS QQQ experiments were collected using Agilent Mass Hunter Workstation and analyzed using the software packages Mass Hunter Qual and Mass Hunter Quant. Results from MHQuant were exported to Excel, then normalized to internal standard, background subtracted and divided by the tissue mass.

Statistical Analysis: Three-factor ANOVA was used throughout the study to analyze the effects of sex, genotype, and diet, with specific differences listed for each method. Where only male mice were use, two-factor ANOVA was used to analyze the effects of genotype and diet. Factorial ANOVA was performed using JMP Pro14 (version 14.0) software with Tukey post-hoc test used for three-factor (sex, genotype, and diet) and two-factor (sex and genotype, sex and diet, genotype and diet) analysis and students t-test used for one-factor (sex, genotype, or diet) analysis. Analysis of combined factors is indicated by “*”. For example, “sex and genotype” is written is sex*genotype.

## 3. Results

### 3.1. PAS Kinase-Deficient Mice Exhibit Increased Respiration When on A Normal Chow (NC) Diet

**Figure 1:**
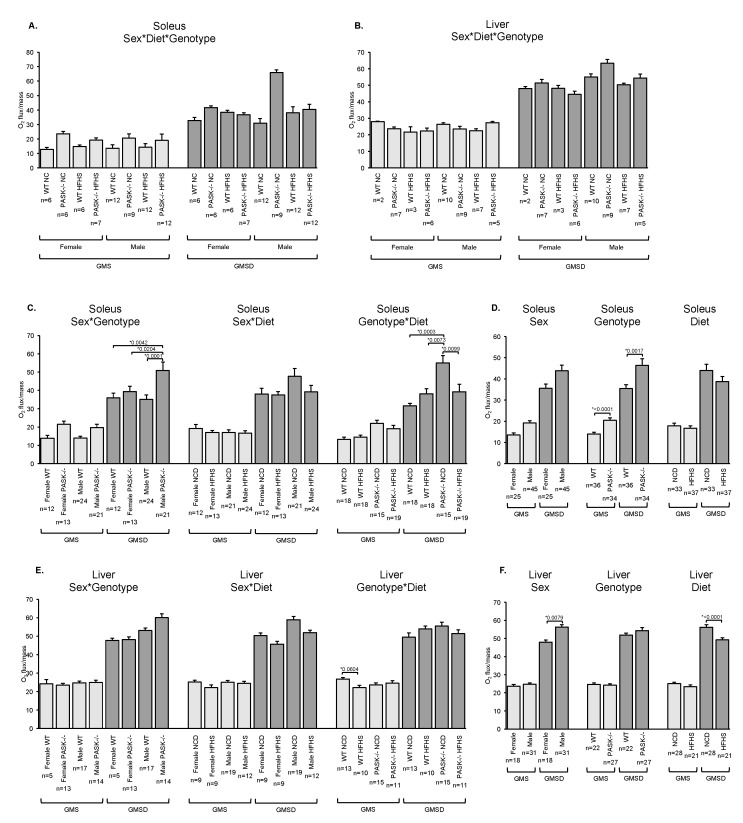
PAS kinase-deficient mice (PASK^−/−^) exhibit increased respiration when on a normal chow diet. (**A**–**F**) Soleus muscle and liver tissue mitochondrial O_2_ consumption determined according to the protocol in Materials and Methods. Results shown are a basal respiration rate (GMS) and oxidative phosphorylation capacity (GMSD). (**A**,**B**) three-factor analysis (sex, genotype, and diet) for (**A**) soleus and (**B**) liver tissue. (**C**,**E**) two-factor analysis (sex and genotype, sex and diet or genotype and diet) for (**C**) soleus and (**E**) liver tissue. (**D**,**F**) one-factor analysis (sex, genotype, or diet) for (**D**) soleus and (**F**) liver tissue. NC is Normal Chow, HFHS is High-Fat High-Sugar diet. For all figures, error bars represent standard error of the mean (SEM). Three-factor ANOVA was performed using JMP Pro14 software. Significant differences were further analyzed by Tukey post-hoc test for three-factor and two-factor comparisons and students t-test for one-factor comparisons. * *p* < 0.05 are reported.

Previous studies have reported a hypermetabolic phenotype for PASK^−/−^ mice on a HF diet, including increased whole-animal O_2_ intake and CO_2_ output [9]. This phenotype may be due to increased respiration in peripheral tissues. Therefore, we measured cellular oxygen consumption rates in female and male soleus muscle, a muscle primarily composed of slow oxidative fibers, as well as in female and male liver tissue (Figure 1). The soleus tissue of both female and male PASK^−/−^ mice displayed a trending increase in basal respiration rate on the NC diet when compared to WT mice upon three-factor (sex, diet, genotype) analysis. Two-factor and one-factor analysis were used to determine if any factors were significantly contributing to this trending increase. Two-factor analysis using combined factors such as sex and genotype (sex*genotype, sex*diet, or genotype*diet) revealed no significant interaction of factors, but further one-factor analysis revealed that the PASK^−/−^ genotype was mainly responsible for significantly increasing respiration (a ~1.5-fold increase), with sex or diet having no significant effect (Figure 1A,D, GMS). In addition, a dramatic effect in oxidative phosphorylation capacity (GMSD) was observed in soleus tissue of the male NC diet PASK^−/−^ mice, which showed a 2-fold increase when compared to the WT in soleus tissue (Figure 1A, GMSD). A three-factor ANOVA followed by two-factor analysis indicate sex*genotype as well as genotype*diet as the main contributing factors to this increase, suggesting a complex interaction of these factors (Figure 1C,D). Combined, the soleus muscle results are consistent with whole-body oxygen consumption increases in PASK^−/−^ mice reported for the whole-animal on a HF diet [9]. In comparison, the liver oxygen consumption rates displayed small differences when compared to the soleus. The WT mice showed decreased basal respiration in response to the HFHS diet, with main dependence on the genotype*diet interaction in the two-factor analysis (Figure 1E). In addition, there were main dependences on sex (an increase in male) and diet (a decrease on HFHS) in the one-factor analysis for liver oxidative phosphorylation capacity (Figure 1F).

Adenosine triphosphate (ATP) assays were performed to determine if the increased capacity for oxidative phosphorylation observed in the soleus tissue of PASK^−/−^ mice translates to increased ATP (Figure S2). Soleus tissue from male mice was used due to the increased respiration rates observed in this tissue on the NC diet; however, no significant differences were observed. This inability to observe a difference may in part be due to the necessity to use mice from a later cohort of the same breeding colony (see Figure S2 legend).

### 3.2. Decreased Complex I Protein is Observed on the HFHS Diet

**Figure 2:**
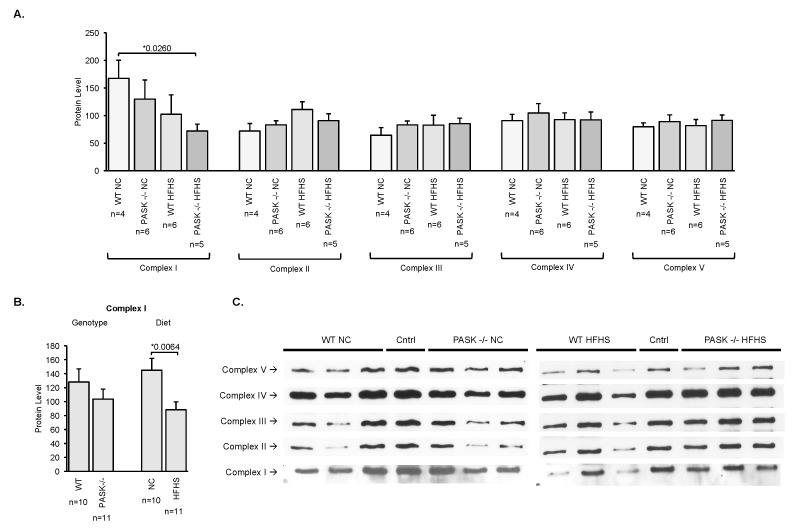
Quantification of 5 electron transport chain complexes using homogenized soleus muscle reveals no significant differences in the PAS kinase-deficient male (PASK^−/−^) mice compared to the WT. (**A**) Soleus tissue was homogenized and analyzed by western blot using the OxPhosBlue Native WB Antibody Cocktail (ThermoFisher Scientific, Waltham, MA, USA) containing mouse monoclonal NDUFA9 (complex I), SDHA (complex II), UQCRC2 (complex III, core II), COX IV (complex IV, subunit IV) and ATP5A (complex V alpha subunit) antibodies. Protein concentration was determined before loading using the Pierce Coomassie Plus (Bradford) Assay Reagent (ThermoFisher Scientific, Waltham, MA, USA). The same control sample (cntrl) was loaded on each gel for normalization between gels. (**B**) Plots of one-factor (genotype or diet) analysis of complex I. (**C**) Representative western blots for each complex are shown. Each biological replicate *n* > 4 was run in duplicate. Error bars represent SEM. Two-factor (genotype and diet) ANOVA was performed using JMP Pro14 software with students t-test performed on significant differences, * *p* < 0.05 is shown.

To investigate the molecular mechanisms behind the increased basal and oxidative phosphorylation capacity in PASK^−/−^ mice soleus tissue, western blot analysis was used to quantify central electron transport chain protein subunits including NDUFA9 (complex I), SDHA (complex II, 70), UQCRC2 (complex III, core II), COX IV (complex IV, subunit IV), and ATP5A (complex V alpha subunit) (Figure 2). Male mice were used due to the increased magnitude of respiration effects observed (see Figure 1). Quantification of these 5 electron transport chain complexes reveals one significant difference in the HFHS PASK^−/−^ mice compared to the NC WT for complex I (Figure 2A). Further one-factor ANOVA analysis revealed that this difference is due to diet and not genotype, with the HFHS diet decreasing complex I (Figure 2B). Thus, the effects of PAS kinase on basal respiration or oxidative phosphorylation capacity in the male mice may not be detectable by our western blot assay. For example, the effects may be on other subunits of these complexes or in other pathways, or they may be post-translational modifications as can often be expected for a protein kinase.

### 3.3. Male PASK^−/−^ Mice Displayed Resistance to Accumulation of Hepatic Triglyceride When Placed on A HFHS Diet

**Figure 3:**
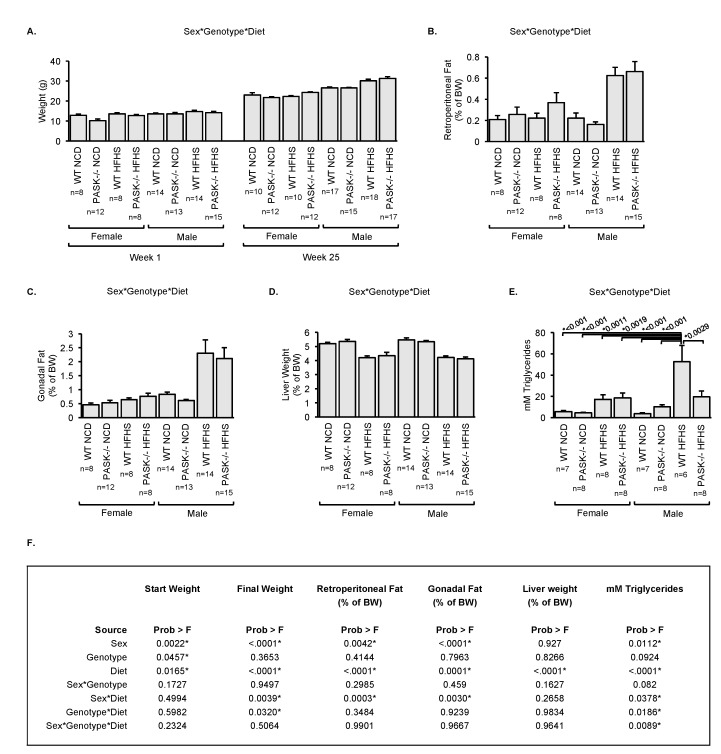
PAS kinase deficiency (PASK^−/−^) protects against HFHS-induced accumulation of hepatic triglycerides. (**A**) Body weight of male and female mice at the start of the diet (week 1, 12-week-old mice) and the end of the diet (week 25). (**B**) Retroperitoneal fat, (**C**) gonadal fat and (**D**) liver weight as a percentage of Body Weight (BW). (**E**) Hepatic triglyceride quantification for female and male mice using BioVision Triglyceride Quantification kit. (**F**) Factorial ANOVA analysis (sex, genotype, and diet) results for the data presented in (**A**–**E**). NC is Normal Chow diet, HFHS is High-Fat High-Sugar diet. For all figures, error bars represent SEM. Three-factor ANOVA was performed using JMP Pro14 software with Tukey post-hoc test for three-factor and two-factor (sex and genotype, sex and diet, or genotype and diet) comparisons and students t-test for one-factor (sex, genotype, or diet) comparisons. * *p* < 0.05 are shown in (**A**–**E**) for the three-factor analysis.

Total body weight was measured over 25 weeks, with fat pads measured at week 25 (Figure 3A–C). Female PASK^−/−^ mice displayed a trend for decreased starting weight (Figure 3A, week 1 of diet) on the standard diet. Further factorial ANOVA analysis revealed the decreased female PASK^−/−^ starting weight was mainly due to independent effects of sex, genotype, and diet (Figure 3F, Figure S3A–C). Male mice weighed significantly more than the female mice after 25 weeks on the HFHS diet. The two-factor interaction analysis suggested these effects were due to interactions between sex*diet and genotype*diet (Figure 3F and Figure S3A–C). No significant difference in weight was observed at 25 weeks when comparing the WT and PASK^−/−^ mice on the HFHS diet. This result is contrary to the resistance to weight gain on a HF diet reported for PASK^−/−^ male mice [9]. These differences could be due to the HF versus HFHS diet, statistical analysis differences, or experimental differences since these were not performed on the same cohort. In male HFHS mice, there was a higher percentage of both retroperitoneal and gonadal fat that was not seen in female mice (Figure 3B,C). The two-factor interaction analysis suggested the main contributing factors to be a significant interaction between sex*diet for both retroperitoneal and gonadal fat, but not sex*genotype or genotype*diet (Figure 3F and Figure S3D–I). Thus, no significance was seen for the PASK^−/−^ genotype. When liver weight was measured as a percentage of body weight, a trending decrease in weight was seen in both male and female mice on a HFHS diet regardless of genotype (Figure 3D). The two-factor interaction revealed no major contributions from interaction, while the one-factor interaction analysis revealed diet as the only main contributing factor (Figure 3F and Figure S3J–L). In summary, the most significant effects on weight due to PAS kinase deficiency appear to be decreased starting weights of female mice. Additionally, females do not display the same magnitude of body weight difference on the HFHS diet compared to the NC diet as the males do, while both male and female on the HFHS diet have decreased relative liver weight.

Both PASK^−/−^ male mice [18] and rats [13] were previously shown to be resistant to hepatic triglyceride accumulation when placed on a HF diet. The effect of PAS kinase in male mice on the HFHS diet appeared to be similar when we assayed total hepatic triglycerides with an enzymatic kit (Figure 3E). Hepatic triglycerides increased in WT male mice placed on the HFHS diet, while no significant increase occurred in PASK^−/−^ male mice on the same diet (there was a 2.7-fold reduction in total triglycerides when compared to the WT mice on the HFHS diet). This trend was not reflected in the female mice, as they appeared to be resistant to hepatic triglyceride accumulation on a HFHS diet. Factorial ANOVA revealed that male WT HFHS mice had elevated triglycerides when compared to all other mouse groups, and indicated this difference was due to a complex, three-factor interaction between sex, genotype, and diet (Figure 3F and Figure S3M–O).

### 3.4. Male PASK^−/−^ Mice Resist Hepatic Triglyceride Accumulation in A Relatively Non-Specific Manner

**Figure 4:**
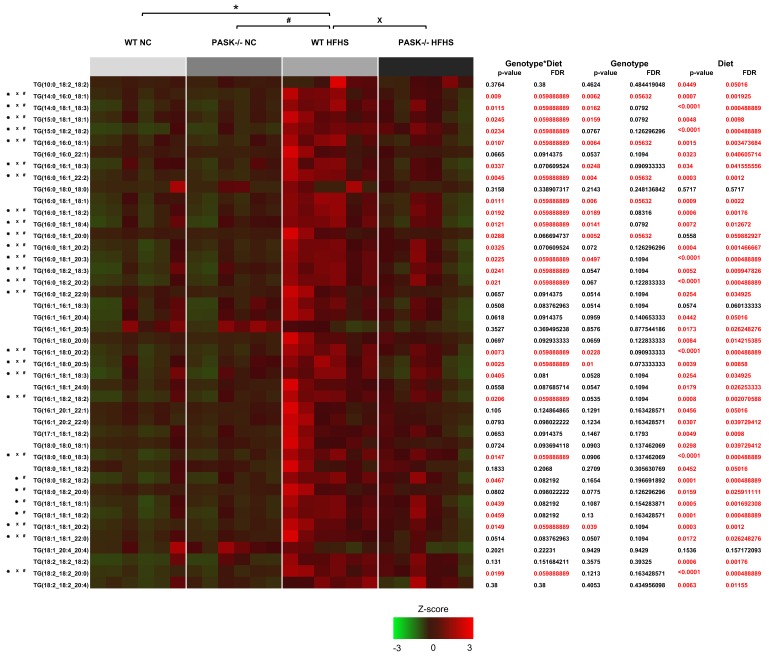
LC/MS triglyceride analysis of WT and PASK^−/−^ male mice on NC and HFHS reveal significant changes in individual triglycerides. A heat map LC/MS triglyceride analysis from male WT and PASK^−/−^ mice placed on a NC and HFHS diet (*n* = 6 for each of 4 sample groups) *, #, or X, *p* < 0.05 when analyzed by two-factor ANOVA and Tukey post-hoc test. Two-factor (genotype and diet) and one-factor (genotype or diet) interaction analysis is provided in a table on the right with *p*-values and false discovery rates (FDR) given. Significant *p*-values (*p* < 0.05) are shown in read, with alternative FDR *q*-value cutoff (*p* < 0.0599) provided in red for comparison.

**Figure 5:**
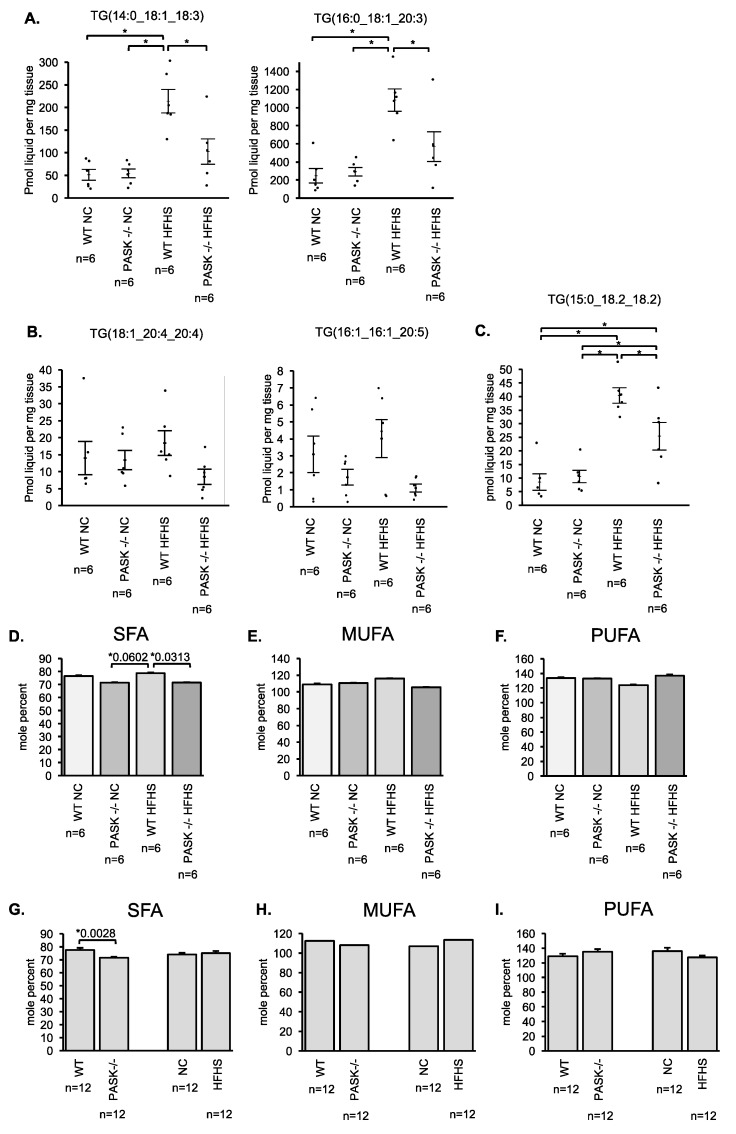
Saturated fatty-acid side chains are elevated in WT male mice on the HFHS diet but not PASK^−/−^ male mice on the HFHS diet. (**A**) Examples of PAS kinase-dependent protection from triglyceride accumulation. (**B**) Examples of triglycerides that were not significantly affected by the HFHS diet. (**C**) One triglyceride that increased in response to the HFHS diet in the PASK^−/−^ mouse. Bars represent SEM. * *p* < 0.05 when analyzed by two-factor ANOVA and Tukey post-hoc test. (**D**–**F**) Data in Figure 4 was analyzed for saturated fatty acid (SFA), monounsaturated fatty acid (MUFA), and polyunsaturated fatty acid (PUFA) side chains within each triglyceride. Side-chain abundance was calculated using mole percent ratio (percentage of moles of each fatty-acid side chain compared to total mole concentration). *p* < 0.1 when analyzed by two-factor ANOVA and Tukey post-hoc test. (**G**–**I**) One-factor (genotype or diet) analysis of (**D**–**F**). *p* < 0.05 when analyzed by student’s t-test.

To determine if the PAS kinase-associated protection from triglyceride accumulation was specific for particular triglycerides, LC/MS lipidomic analysis was performed on 6 male mice from each of the 4 mouse groups by the University of Utah Metabolomics Core Facility. When all 44 of the triglycerides analyzed were totaled the mice displayed a similar pattern to the enzymatic total triglyceride quantification performed in our laboratory (which may represent far more than 44 triglycerides). An analysis of each of the triglycerides quantified in the LC/MS lipidomic study is presented in a heat map (Figure 4A). PAS kinase appears to regulate triglycerides almost indiscriminately, with the pattern of most triglycerides mimicking that which was seen in the total triglycerides. Twenty-five triglycerides were significantly elevated in the WT mice (*p* < 0.05) but not the PASK^−/−^ mice in response to HFHS diet, 10 others are close to significance (*p* < 0.1). Examples of 2 of these 25 triglycerides are shown (Figure 5A). The false discovery rate (FDR) *q*-value was less than 8.22% for all these 25 indicating that ~2 (2.05) may be false positives. To achieve a predicted rate of only one false positive, a FDR *q*-value of <0.06 must be chosen, leaving 18 triglycerides as significant. A few triglycerides were not elevated in WT HFHS mice, suggesting that diet did not affect the accumulation of these triglycerides. Examples from 2 of these non-affected triglycerides are shown (Figure 5B). One triglyceride to note is TG (15:0_18.2_18.2) (Figure 5C). This triglyceride is elevated in the WT HFHS as can be expected, but it is also significantly elevated in the PASK^−/−^ HFHS. When the side chains of the 44 triglycerides were analyzed and quantified as saturated fatty acid (SFA), monounsaturated fatty acid (MUFA), and polyunsaturated fatty acid (PUFA), PAS kinase appeared to more specifically regulate SFA (Figure 5D–I). The two-factor interaction analysis indicated that the SFA significance was mainly due to an interaction between diet and genotype. In support of this effect, the 25 PAS kinase-dependent triglycerides (*q*-value < 0.0822%) represented 90% of the 44 triglycerides by abundance. 

## 4. Discussion

The regulation of respiratory and triglyceride metabolism lies at the center of several prevalent diseases including heart disease, obesity, diabetes, metabolic syndrome, and cancer. Previous studies have shown PASK^−/−^ mice to be resistant to hepatic triglyceride accumulation and to have a whole-body hypermetabolic phenotype when placed on a HF diet [18]. In support of these observations, we have shown PAS kinase to play a pivotal role in regulating cellular respiration in yeast as well [25,26]. Herein we build upon these studies by performing the first characterization of cellular respiration, quantification and analysis of hepatic triglyceride accumulation, and body weight changes in both male and female PASK^−/−^ mice (previous reports were solely in male mice). Furthermore, this is the first report of these mice on a HFHS diet, a diet that may more accurately reflect the western diet [22]. PAS kinase has been previously shown to be activated by high glucose in mammalian tissue [14]. Due to the presence of sugar in this HFHS diet, we expected PAS kinase to be more active and the related phenotypes to be more pronounced.

We observed increased oxygen consumption rates in PASK^−/−^ mice in soleus muscle tissue that was dependent on genotype (Figure 1). This is consistent with the whole-body hypermetabolism (increased O_2_ uptake and CO_2_ output) in PASK^−/−^ mice placed on a HF diet [18] as well as the increased cellular respiration rate in PAS kinase-deficient yeast [25,26]. This increase, however, was often seen on the NC diet and was blunted on HFHS (Figure 1). Male PASK^−/−^ mice also displayed significant increases in the oxidative phosphorylation capacity in soleus tissue. ANOVA analysis revealed this difference to be mainly due to complex interactions between sex*genotype as well as genotype*diet. Overall, the effects of PAS kinase on respiratory function were detectable on the NC diet but not the HFHS, in contrast to the whole-body hypermetabolic phenotype reported on the HF diet [18]. This result is opposite of what we expected due to previous reports of PAS kinase activation by high glucose [14]. These differences may be due to many experimental variables including single cell respiration versus whole-animal metabolism, feed and the metabolism of feed, age of animal, in vitro versus in vivo respiration assay conditions, generations from F0 parent, compensation by alternate pathways, etc. However, the trend of PASK^−/−^ displaying increased metabolism is conserved wherever we do see a difference in respiration. 

In contrast to the respiratory function, the role of PAS kinase in regulating hepatic triglyceride accumulation is most evident in male mice on the HFHS diet (Figure 3E). The WT male mice displayed a dramatic increase in hepatic triglyceride levels when placed on the HFHS diet, whereas the PASK^−/−^ male mice did not show any appreciable increase. This effect was similar to that reported for the PASK^−/−^ mice on the HF diet [18]. The female mice, in contrast, appeared to be more resistant to liver triglyceride accumulation in response to the HFHS diet (Figure 3E). Our results are consistent with previous reports that female WT mice are more resistant to triglyceride accumulation on the HFHS diet [27]. This suggests that sex differences may be overshadowing the role of PAS kinase in females.

LC/MS lipidomic analysis of 44 hepatic triglycerides from male mice revealed that almost all (40/44) were elevated in response to the HFHS diet in WT mice (*p* < 0.05). PASK^−/−^ mice were resistant to accumulation of 25 of these 40 triglycerides (*p* < 0.05, FDR *q*-value < 0.0822), with another 10 close to significance. ANOVA analysis revealed that this resistance was mainly due to the interaction of genotype*diet rather than sex*diet or diet*genotype. Further analysis of these 44 triglycerides revealed that PAS kinase preferentially regulates saturated fatty-acid side chains (SFA) (Figure 5D–I). The 25 PAS kinase-dependent triglycerides represented 90% of the 44 triglycerides by abundance, indicating the side-chain analysis of all 44 may reveal PAS kinase-dependent effects.

As predicted from the triglyceride data, male mice displayed an increase in total body weight as well as retroperitoneal and gonadal fat pad (% of body weight) on the HFHS diet, while we saw no significant increase in female mice (Figure 3). However, PAS kinase deficiency did not affect body weight or fat pad weight as it did triglyceride accumulation. When looking at the combined data for female mice (which displayed only very small changes in total body mass, fat accumulation, or hepatic triglyceride accumulation) it appears that the HFHS diet is having a very limited effect on female metabolism. This study highlights how diet and genetic effects may be masked by sex differences, making it critical to continue studies of PAS kinase in both male and female mice to truly understand how it is functioning in both sexes.

PAS kinase has been shown to be activated by high glucose in cultured human pancreatic islet cells [14] and to be regulated by glucose levels in yeast as well [28], stimulating our interest in the HFHS diet which more closely approximates the western diet and tends to abundant in high fructose corn-syrup and milk products [22]. In this study, the respiratory effects observed in PASK^−/−^ versus WT mice occurred on the NC diet, whereas the triglyceride effects were most significant on the HFHS diet. In addition to the experimental variabilities discussed above, these results may indicate differential regulation of PAS kinase functions, consistent with protein kinases regulating tens of substrates with alternate functions. They may also reflect redundant pathways that mask PAS kinase deficiency, or genetic adaptations that have occurred in these knockout mice [29,30,31]. Such adaptations are less likely to be obscuring our triglyceride data because pharmacological PAS kinase inhibition has also been shown to protect against HF diet-inducted hepatic triglyceride accumulation in rats [13].

The molecular substrates of PAS kinase that elicit the effects on respiration and triglyceride accumulation are just beginning to be uncovered. Wu et al. recently reported PAS kinase as essential for SREBP-1c maturation in cultured hepatic cells [13]. SREBP-1 is clearly an important transcription factor that drives hepatic fatty acid and triglyceride biosynthesis (for reviews see [32,33,34] and articles therein); however, it is not the only one. Upstream stimulation factors (USFs) are bHLH-leucine zipper transcription factors that bind as homo- or heterodimers to promoter E boxes with the DNA sequence CANNTG and have been associated with hyperlipidemia in many studies [35,36,37,38,39,40,41,42,43,44,45,46,47,48]. Several studies have provided evidence for the direct regulation of fatty-acid synthase expression by USF1 and USF2 [49,50,51]. We have recently provided evidence that PAS kinase phosphorylates and inhibits the yeast homolog of USF1 (Cbf1), which in turn controls both cellular respiration and lipid biosynthesis in yeast [25,26]. Its mammalian homolog, USF1, complements the respiratory defect of CBF1-deficient yeast and is phosphorylated by hPASK in vitro [26]. USF1 and SREBP-1 have been reported to have both synergistic, direct binding [52,53] and independent modes of action [51,54], thus PAS kinase may control triglyceride biosynthesis through the regulation of both proteins. Our ongoing studies are focused on the role of PAS kinase in regulating USF1 function in mammalian cells, including both respiratory and lipid roles.

## 5. Conclusions

Herein we investigated the effects of a HFHS diet, sex and PAS kinase-deficiency on respiratory metabolism, body, fat and liver weight, as well as hepatic triglyceride accumulation. PAS kinase-deficiency resulted in increased basal respiration (~1.5-fold) in soleus tissue, and for male mice, an increased oxidative phosphorylation capacity (2-fold) when on a NC diet. For WT mice, the HFHS diet decreased basal respiration in liver tissue. Although PAS kinase-deficiency did not appear to protect male mice from weight gain, it did decrease hepatic triglyceride accumulation significantly on the HFHS diet, with PASK^−/−^ male mice having a 2.7-fold decrease when compared to the WT mice. Female mice appeared to be protected from both weight gain and liver triglyceride accumulation on the HFHS diet, which may mask the effects of PAS kinase in these pathways. These results solidify PAS kinase as a regulator of cellular respiration and hepatic triglyceride accumulation, phenotypes associated with diabetes and metabolic syndrome.

## Supplementary Materials

The following are available online at http://www.mdpi.com/2072-6643/10/12/1990/s1, Figure S1: An account of all mice used in this study including mouse number for this study, genotype, sex, diet, Oxygen Consumption Rates in soleus tissue (OCR soleus), Oxygen Consumption Rates in liver tissue (OCR liver), electron chain western blot (ETC WB), week 1 and week 25 BW (Body Weight), Liver Weight, Retroperitoneal Fat weight (RetroFat), Gonadal Fat weight, total triglycerides (Total TG), triglyceride mass spectrometry (TG MS). Figure S2: ATP levels of soleus tissue isolated from WT and PASK^−/−^ mice on a normal chow (NC) or high fat high sugar (HFHS) diet suggest no significant differences. Figure S3: Figures of analysis for three-factor ANOVA of mouse body weights and triglycerides.
