# Supplementary material for: Per-Arnt-Sim Kinase (PASK) Deficiency Increases Cellular Respiration on a Standard Diet and Decreases Liver Triglyceride Accumulation on a Western High-Fat High-Sugar Diet
Source: Nutrients. 2018 Dec 15;10(12):1990. doi: 10.3390/nu10121990 (PMC6316003; doi:10.3390/nu10121990)
Supplement: Supplementary file 1 [file nutrients-10-01990-s001.pdf]

Mouse Number	Sex	Genotype	Diet	OCR Soleus	OCR Liver	ETC WB	Week 1 BW	Week 25 BW	Retro Fat	Gonadal Fat	Liver Weight	Total TG	TG MS
4-3	F	WT	NC	+	-	-	-	+	-	-	-	-	-
9-3	F	WT	NC	+	-	-	-	+	-	-	-	-	-
32-7	F	WT	NC	+	-	-	+	+	+	+	+	-	-
44-5	F	WT	NC	+	+	-	+	+	+	+	+	+	-
46-1	F	WT	NC	+	-	-	+	+	+	+	+	+	-
46-6	F	WT	NC	+	-	-	+	+	+	+	+	+	-
60-1	F	WT	NC	-	-	-	+	+	+	+	+	+	-
60-6	F	WT	NC	-	-	-	+	+	+	+	+	+	-
71-2	F	WT	NC	-	+	-	+	+	+	+	+	+	-
84-4	F	WT	NC	-	-	-	+	+	+	+	+	+	-
20-4	F	PASK -/-	NC	+	-	-	+	+	+	+	+	-	-
25-2	F	PASK -/-	NC	+	-	-	+	+	+	+	+	+	-
25-3	F	PASK -/-	NC	+	-	-	+	+	+	+	+	-	-
34-5	F	PASK -/-	NC	+	-	-	+	+	+	+	+	-	-
41-3	F	PASK -/-	NC	+	+	-	+	+	+	+	+	+	-
45-8	F	PASK -/-	NC	+	+	-	+	+	+	+	+	+	-
46-8	F	PASK -/-	NC	-	-	-	+	+	+	+	+	+	-
68-1	F	PASK -/-	NC	-	+	-	+	+	+	+	+	-	-
71-7	F	PASK -/-	NC	-	+	-	+	+	+	+	+	+	-
76-3	F	PASK -/-	NC	-	+	-	+	+	+	+	+	+	-
77-1	F	PASK -/-	NC	-	+	-	+	+	+	+	+	+	-
77-4	F	PASK -/-	NC	-	+	-	+	+	+	+	+	+	-
13-4	F	WT	HFHS	+	-	-	-	+	-	-	-	-	-
28-6	F	WT	HFHS	+	-	-	+	+	+	+	+	+	-
32-4	F	WT	HFHS	+	-	-	-	+	-	-	-	-	-
33-7	F	WT	HFHS	+	-	-	+	+	+	+	+	+	-
41-1	F	WT	HFHS	+	+	-	+	+	+	+	+	+	-
44-3	F	WT	HFHS	+	+	-	+	+	+	+	+	+	-
53-6	F	WT	HFHS	-	-	-	+	+	+	+	+	+	-
57-2	F	WT	HFHS	-	+	-	+	+	+	+	+	+	-
86-3	F	WT	HFHS	-	-	-	+	+	+	+	+	+	-
88-4	F	WT	HFHS	-	-	-	+	+	+	+	+	+	-
7-7	F	PASK -/-	HFHS	+	-	-	-	+	-	-	-	-	-
19-3	F	PASK -/-	HFHS	+	-	-	-	+	-	-	-	-	-
21-6	F	PASK -/-	HFHS	+	-	-	-	+	-	-	-	-	-
44-7	F	PASK -/-	HFHS	+	+	-	+	+	+	+	+	+	-
45-6	F	PASK -/-	HFHS	+	+	-	-	+	-	-	-	-	-
45-9	F	PASK -/-	HFHS	+	+	-	+	+	+	+	+	+	-
46-4	F	PASK -/-	HFHS	+	-	-	+	+	+	+	+	+	-
53-3	F	PASK -/-	HFHS	-	-	-	+	+	+	+	+	+	-
54-5	F	PASK -/-	HFHS	-	+	-	+	+	+	+	+	+	-
57-1	F	PASK -/-	HFHS	-	+	-	+	+	+	+	+	+	-
76-1	F	PASK -/-	HFHS	-	+	-	+	+	+	+	+	+	-
98-7	F	PASK -/-	HFHS	-	-	-	+	+	+	+	+	+	-
4-4	M	WT	NC	+	-	-	-	+	-	-	-	+	-
8-3	M	WT	NC	+	-	-	-	+	-	-	-	+	+
9-2	M	WT	NC	+	-	-	-	+	-	-	-	+	-
17-2	M	WT	NC	+	-	+	+	+	+	+	+	+	-
18-1	M	WT	NC	+	-	-	+	+	+	+	+	+	-
21-2	M	WT	NC	+	-	-	+	+	+	+	+	+	+
31-6	M	WT	NC	+	-	-	+	+	+	+	+	+	+
40-4	M	WT	NC	+	-	-	+	+	+	+	+	-	-
42-7	M	WT	NC	+	+	+	+	+	+	+	+	-	+
43-7	M	WT	NC	+	+	-	+	+	+	+	+	-	-
46-3	M	WT	NC	+	+	-	+	+	+	+	+	-	-
46-7	M	WT	NC	+	+	-	+	+	+	+	+	-	+
55-5	M	WT	NC	-	+	-	+	+	+	+	+	-	-
58-1	M	WT	NC	-	+	+	+	+	+	+	+	-	-
59-4	M	WT	NC	-	+	-	-	-	-	-	-	-	-
63-1	M	WT	NC	-	+	+	+	+	+	+	+	-	-
76-2	M	WT	NC	-	+	-	+	+	+	+	+	-	+
77-2	M	WT	NC	-	+	-	+	+	+	+	+	-	-
5-9	M	PASK -/-	NC	+	-	-	-	+	-	-	-	+	-
9-5	M	PASK -/-	NC	+	-	-	-	+	-	-	-	-	+
32-1	M	PASK -/-	NC	+	-	-	+	+	+	+	+	+	-
34-4	M	PASK -/-	NC	+	-	+	+	+	+	+	+	-	-
36-1	M	PASK -/-	NC	+	-	-	+	+	+	+	+	-	-
38-5	M	PASK -/-	NC	+	-	-	+	+	+	+	+	-	+
41-5	M	PASK -/-	NC	+	+	-	+	+	+	+	+	-	-
43-2	M	PASK -/-	NC	+	+	+	+	+	+	+	+	-	+
44-4	M	PASK -/-	NC	+	+	+	+	+	+	+	+	-	-
55-6	M	PASK -/-	NC	-	+	-	+	+	+	+	+	+	+
62-1	M	PASK -/-	NC	-	+	+	+	+	+	+	+	+	-
68-5	M	PASK -/-	NC	-	+	+	+	+	+	+	+	+	-
75-1	M	PASK -/-	NC	-	+	+	+	+	+	+	+	+	-
75-3	M	PASK -/-	NC	-	+	-	+	+	+	+	+	+	+
75-6	M	PASK -/-	NC	-	+	-	+	+	+	+	+	+	+
7-1	M	WT	HFHS	+	-	-	-	+	-	-	-	+	-
8-1	M	WT	HFHS	+	-	-	-	+	-	-	-	+	+
8-4	M	WT	HFHS	+	-	-	-	+	-	-	-	+	-
12-1	M	WT	HFHS	+	-	+	-	+	-	-	-	+	-
14-1	M	WT	HFHS	+	-	-	+	+	+	+	+	-	+
18-4	M	WT	HFHS	+	-	-	+	+	+	+	+	+	-
21-4	M	WT	HFHS	+	-	-	+	+	+	+	+	+	+
22-1	M	WT	HFHS	-	-	-	+	+	+	+	+	-	-
23-3	M	WT	HFHS	+	-	-	+	+	+	+	+	-	-
28-7	M	WT	HFHS	+	-	-	+	+	+	+	+	-	+
41-2	M	WT	HFHS	+	+	+	+	+	+	+	+	-	-
42-3	M	WT	HFHS	+	+	-	+	+	+	+	+	-	-
45-4	M	WT	HFHS	+	+	-	+	+	+	+	+	-	+
61-1	M	WT	HFHS	-	-	+	+	+	+	+	+	-	-
63-3	M	WT	HFHS	-	+	+	+	+	+	+	+	-	-
69-2	M	WT	HFHS	-	+	+	+	+	+	+	+	-	-
71-6	M	WT	HFHS	-	+	-	+	+	+	+	+	-	-
77-6	M	WT	HFHS	-	+	+	+	+	+	+	+	-	+
5-2	M	PASK -/-	HFHS	+	-	-	-	+	-	-	-	+	-
5-5	M	PASK -/-	HFHS	+	-	-	-	+	-	-	-	+	+
14-2	M	PASK -/-	HFHS	+	-	-	+	+	+	+	+	+	+
19-2	M	PASK -/-	HFHS	+	-	+	+	+	+	+	+	+	-
21-3	M	PASK -/-	HFHS	+	-	+	+	+	+	+	+	-	-
21-7	M	PASK -/-	HFHS	+	-	+	+	+	+	+	+	-	-
23-1	M	PASK -/-	HFHS	+	-	+	+	+	+	+	+	-	-
31-5	M	PASK -/-	HFHS	+	-	-	+	+	+	+	+	-	-
35-1	M	PASK -/-	HFHS	+	-	-	+	+	+	+	+	-	-
36-2	M	PASK -/-	HFHS	+	-	-	+	+	+	+	+	-	-
38-6	M	PASK -/-	HFHS	+	-	-	+	+	+	+	+	-	-
43-8	M	PASK -/-	HFHS	+	+	-	+	+	+	+	+	-	-
59-3	M	PASK -/-	HFHS	-	+	+	+	+	+	+	+	-	-
62-6	M	PASK -/-	HFHS	-	+	-	+	+	+	+	+	+	+
72-6	M	PASK -/-	HFHS	-	+	-	+	+	+	+	+	+	+
75-2	M	PASK -/-	HFHS	-	-	-	+	+	+	+	+	+	+
75-7	M	PASK -/-	HFHS	-	+	-	+	+	+	+	+	+	+

Figure S1. An account of all mice used in this study including mouse number for this study, genotype, sex, diet, Oxygen Consumption Rates in soleus tissue (OCR soleus), Oxygen Consumption Rates in liver tissue (OCR liver), electron chain western blot (ETC WB), week 1 and week 25 BW (Body Weight), Liver Weight, Retroperitoneal Fat weight (RetroFat), Gonadal Fat weight, total triglycerides (Total TG), triglyceride mass spectrometry (TG MS). Mice from supplementary Figure 2 are not included because they are not otherwise used in the study.

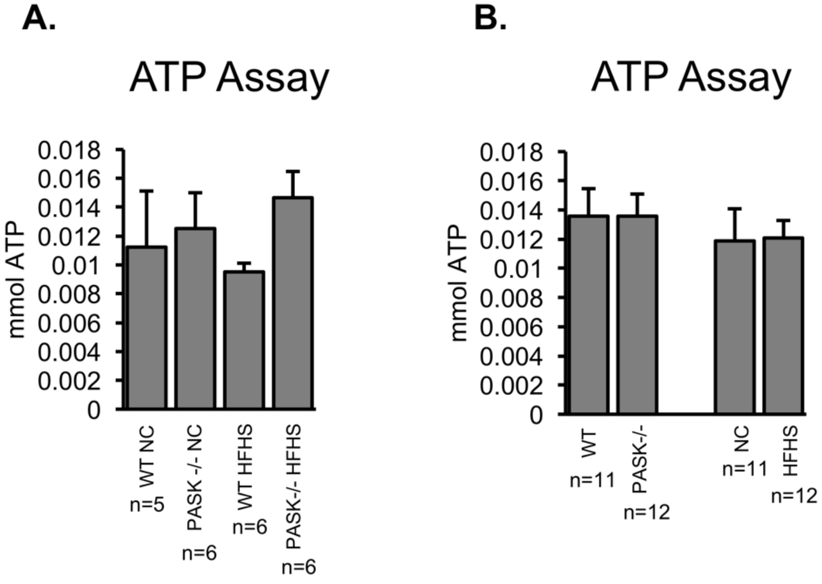

Source	Prob > F
Genotype	0.2141
Diet	0.9375
Genotype*Diet	0.4414

Figure S2. ATP levels of soleus tissue isolated from WT and PASK$^{-/-}$ mice on a normal chow (NC) or high fat high sugar (HFHS) diet suggest no significant differences. Mice were from the same breading colony but an alternate immunology study that had the same genotypes and treatments due to technical difficulties with variability in the flash frozen samples (samples were originally flash frozen for use in both western blot or ATP assay). To overcome these technical variabilities, soleus muscle was harvested and stored at -80°C in ATP Assay buffer (Sigma-Aldrich Adenosine 5'-triphosphate (ATP) Bioluminescent Assay Kit (FLAA-1KT)) prior to the assay. Soleus muscle was homogenized using the Bullet Blender Storm 24 (Next Advance) with 2mm Zirconium oxide beads then deproteinized using the BioVision Deproteinizing Sample Preparation Kit (K808). Due to small amount of protein (0.01ug/ul) the PCA and neutralization buffer in the kit were diluted (1:10 and 1:20 respectively) to prevent excessive PCA or neutralization buffer being added. ATP assays were run using the Sigma-Aldrich Adenosine 5'-triphosphate (ATP) Bioluminescent assay kit (FLAA-1KT) according to manufacturer's protocol. Luminescence was measured using the BioTek Synergy HT Multi-mode microplate reader. Protein concentration was determined using the Pierce Coomassie Plus (Bradford) Assay Reagent (ThermoFisher Scientific, catalog number 23236).

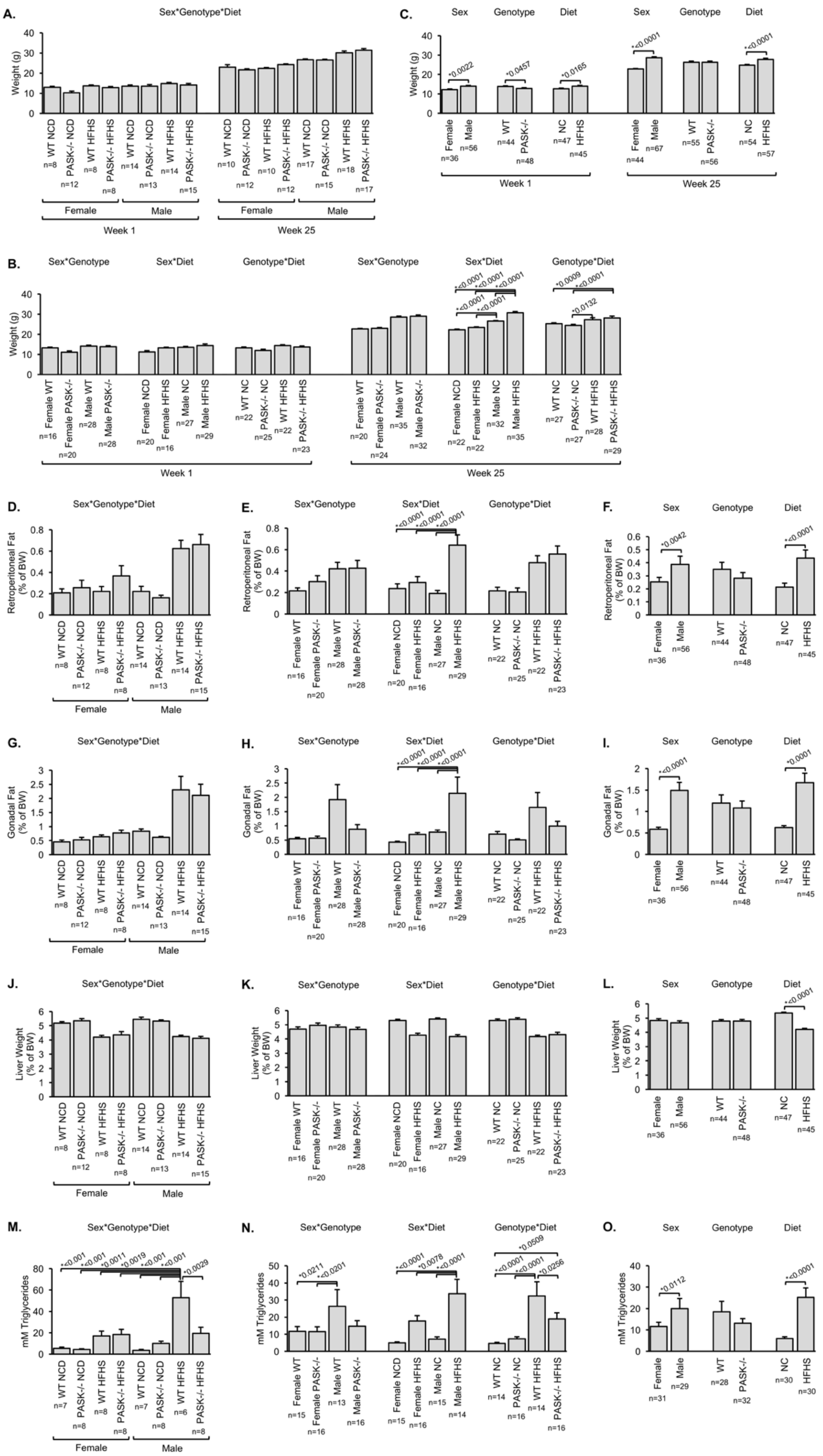
A.
Sex*Genotype*Diet
Weight (g)
WT NCD
PASK-/- NCD
WT HFHS
PASK-/- HFHS
Female
Male
Week 1
Week 25
B.
Sex*Genotype
Sex*Diet
Genotype*Diet
*<0.0001
*0.0009
*0.0132
C.
Sex
Genotype
Diet
*0.0022
*0.0457
*0.0165
*<0.0001
D.
Retroperitoneal Fat (% of BW)
E.
F.
*0.0042
G.
Gonadal Fat (% of BW)
H.
I.
J.
Liver Weight (% of BW)
K.
L.
M.
mM Triglycerides
*<0.001
*0.0011
*0.0019
*0.0029
N.
*0.0211
*<0.0201
*0.0078
*0.0509
*0.0256
O.
*0.0112

Figure S3. Figures of analysis for three-factor ANOVA of mouse body weights and triglycerides. (A) Body weight of male and female mice at the start of the diet (week 1, 12-week-old mice) and the end of the diet (week 25). Retroperitoneal fat (D), Gonadal fat (G) and Liver weight (J) as a percentage of body weight (BW). (M) Hepatic triglyceride quantification for male and female mice using BioVision Triglyceride Quantification kit. (B, E, H, K, N) two-way interaction analysis of (A, D, G, J, M) respectively. (C, F, I, L,O) one-factor analysis of (A,D, G,J, M). NC is Normal Chow diet, HFHS is High-Fat High-Sugar diet. For all figures, error bars represent SEM. Three-factor ANOVA was performed using JMP Pro14 software with Tukey post-hoc test for three-factor and two-factor comparisons and students t-test for one-factor comparisons. *$p < 0.05$ is shown.
